# Proliferation of the WReN spider, an instrument to measure health professionals’ experience of research: a bibliographic study

**DOI:** 10.1186/s12909-019-1693-9

**Published:** 2019-07-09

**Authors:** Lidia Luna Puerta, Christian Apfelbacher, Helen Smith

**Affiliations:** 1 Family Medicine and Primary Care, Lee Kong Chian School of Medicine, Nanyang Technological University Singapore, Singapore, Singapore; 2 Medical Sociology, Institute of Epidemiology and Preventive Medicine, University of Regensburg, Regensburg, Germany; 3 Institute of Social Medicine and Health Economics, Otto von Guericke University Magdeburg, Magdeburg, Germany

**Keywords:** Bibliometrics, Research skills, Research experience, Research culture, Health care professionals

## Abstract

**Background:**

In 1997 the “Wessex Research Network (WReN) Spider” was developed and validated to assess the research experience of general practice based researchers. This bibliometric study traces the use and development of this instrument over 15 years.

**Methods:**

We performed a bibliographic search to identify all the citations of the original article since 2002.

**Results:**

Thirty one relevant papers were found. Publications were classified according to whether they used (*N* = 18) or cited (*N* = 13) the WReN Spider. The majority of these papers came from Australia (N = 18) and 10 papers focussed on the research training of Allied Health Professionals. The WReN Spider was used in 12 studies to assess baseline experience before a training intervention or to compare before and after training scores. The WReN Spider was often (*N* = 9) modified to additionally assess interest, confidence or interest in up-skilling in each of its 10 limbs. It was also often (*N* = 14) used in tandem with open ended questions to gain a more detailed understanding of people’s research skills, or with additional questions focussing on the research context, culture and team. None of the papers confirmed the validation of the WReN Spider, although it was applied in contexts that differed from the one in which it was developed.

**Conclusions:**

The WReN Spider continues to be used to measure the research experience of health care practitioners, but it is frequently enhanced with other questions to look at the wider issues of research success, including collaborators, resource and environment.

## Background

Primary care research aims to improve the quality, effectiveness and cost effectiveness of primary care, in all its manifestations. An essential outcome of this research is to provide the evidence base that primary care services previously lacked [1]. While greater emphasis is given to translational research, and numerous organizations provide support to build and support research capacity in the field of health research [2], factors that influence a positive research culture in health professionals are not fully understood [3]. Different instruments have emerged to assess the topics where professionals’ skills are most lacking and for which priority must be given in the design of appropriate training courses and other capacity building initiatives [2, 4].

**Fig. 1:**
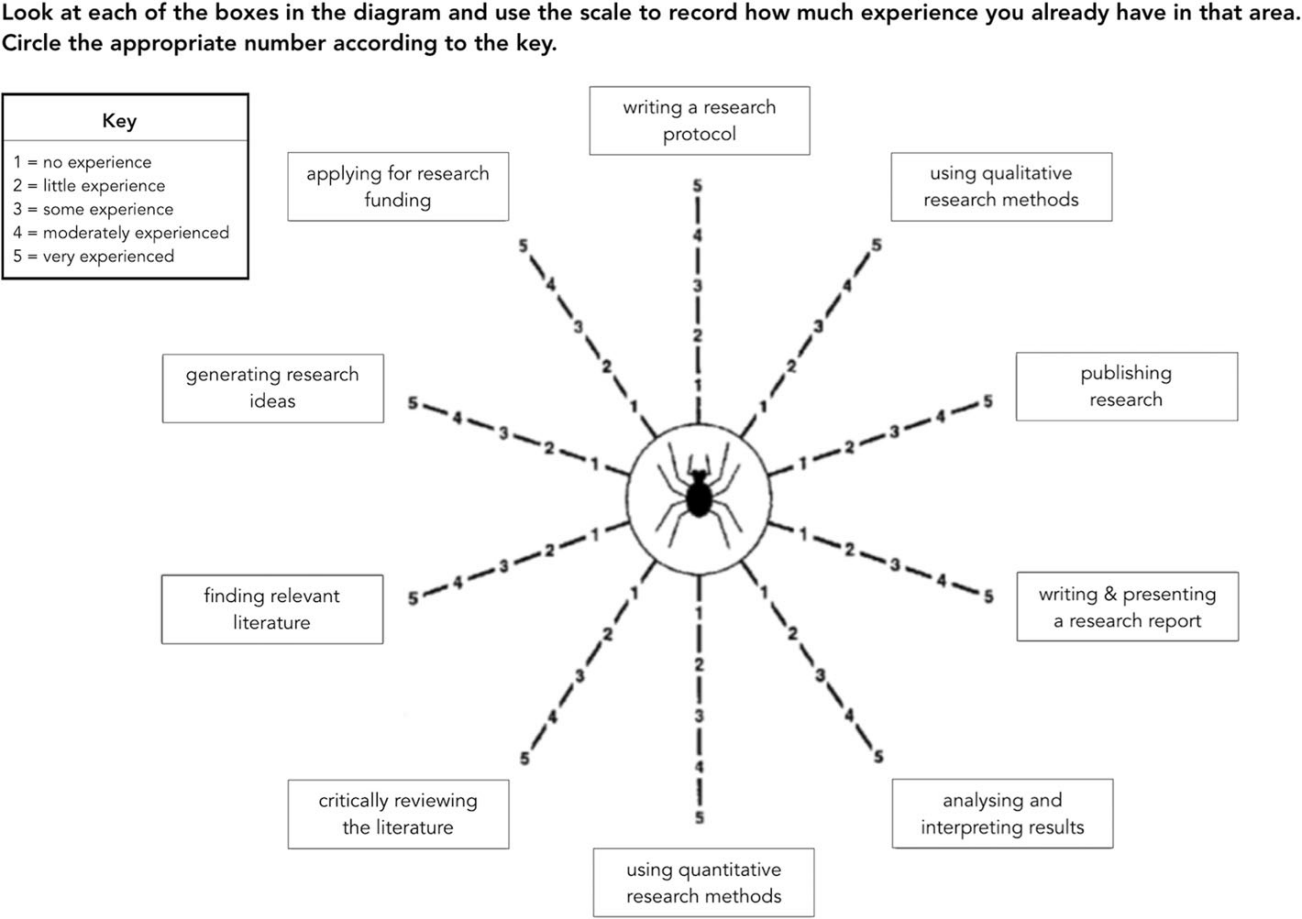
The original WReN Spider as published in 2002 by Smith [5]

The “Wessex Research Network Spider (WReN) Spider” was validated in 1997 as a simple and efficient way of assessing research experience across a large multiprofessional health care group [5]. The WReN Spider, shown in Fig. 1, was designed to assess research experience in order to inform the planning of healthcare professional’s educational programs. Users rate their research experience from 1 = ‘no experience’ to 5 = ‘very experienced’ on 10 limbs (items) relating to discrete components of the research process, including writing a research protocol, analysing and interpreting research results and publishing research. It is unclear to what extent other researchers have adopted and/or possibly adapted the original WReN Spider.

Therefore, the aim of this study was to systematically review the papers which cited the original WReN Spider publication in order to investigate the uses and developments of the WReN Spider by the research community over a period of 15 years (2002 to 2017) since its development.

## Methods

A literature search was conducted in February 2018 on Google Scholar, Scopus and Web of Science to identify all the citations of the original article [5] since its publication in the year 2002. These science citation indexing services were chosen as they provide comprehensive citation searches in different academic and scientific disciplines coupled with ease of accessibility and wide use [6–8]. In Web Of Science, the “Cited Reference Search” function was used, using the title of the original publication to search. In Scopus, the “Search Article Title” option was used, and then the function “View cited by”. The same protocol was used in Google Scholar. The search results from all three searches were collated in EndNote. To ensure we had captured all relevant articles we expanded our search with citation chaining searching backwards and forwards in time for materials that are cited by and also cited the articles we had already identified.

We searched for papers in English and those languages where we had the skills to translate within the research team (German, Spanish, French, Italian, Mandarin, Swedish, Italian, Croatian, Burmese). Papers were included if the original WReN Spider publication [5] appeared in the list of references.

We plotted the publications by year and by place before classifying them according to whether they were examples of (i) utilisation of the WReN Spider in its original or an adapted form, or (ii) merely a citation of the original WReN Spider article.

For papers reporting on (i) use of the WReN Spider we extracted the following:

- Study setting (country & institution e.g. primary care research network)
- Target audience (profession, number)
- Study design (e.g. before / after evaluation, survey)
- Type of use (e.g alone or in conjunction with other instruments, original WReN Spider or modified WReN)
  1. original Spider alone original
  2. original Spider in conjunction with another assessment
  3. modified Spider alone
  4. modified Spider in conjunction with another assessment
- Study findings with respect to Spider measured research experience.

For papers which also adapted the WReN Spider, in addition to the above information, we also extracted the following:

- Characteristics of modified instrument (conceptual model (if any), number of items, number of domains)
- Validity of modified instrument.

For the papers that (ii) cited rather than used the WReN Spider in its original or an adapted form we provide a narrative summary of these.

## Results

**Fig. 2:**
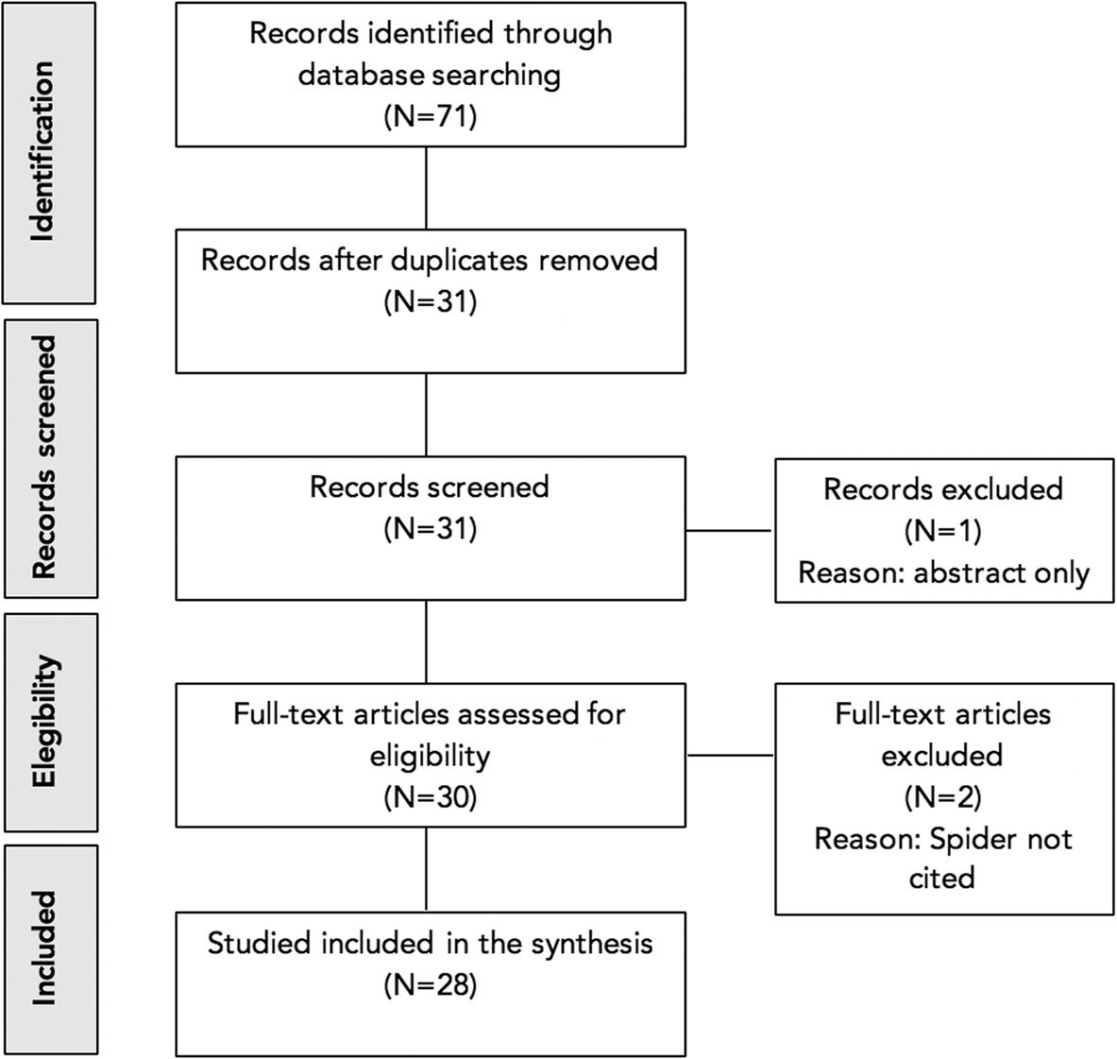
Study flow diagram indicating number of publications screened and finally included

Of the 71 publications retrieved, 32 were found on Google Scholar, whilst the search in Scopus yielded 20 and the Web Of Science yielded 19 publications. In total 31 distinct papers were identified after duplicates were removed (Fig. 2). One paper was indexed in both French and English, so the French duplicate was removed. Two papers had no mention of the WReN Spider in the full-text and therefore were excluded. A further citation was excluded because it was an abstract rather than a full-text publication. Twenty eight papers were included in the final analysis (Fig. 2).

**Fig. 3:**
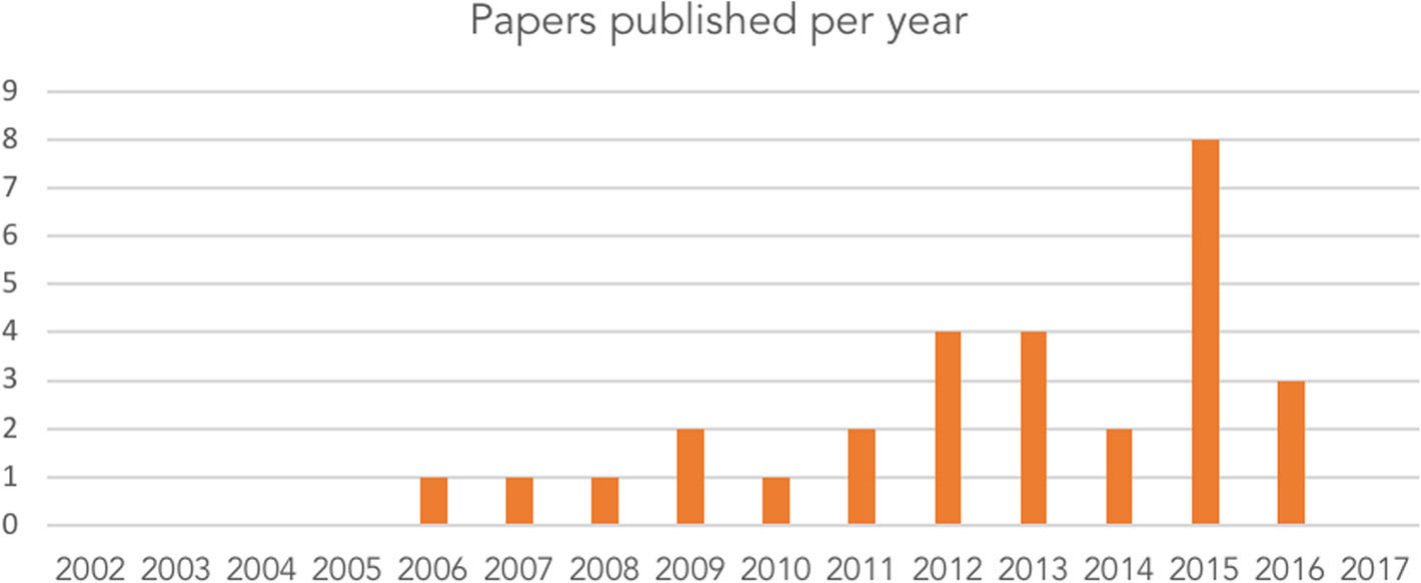
Number of research publications using or citing the WReN Spider per year between 2002 and 2017 (at January 18th, 2018)

**Fig. 4:**
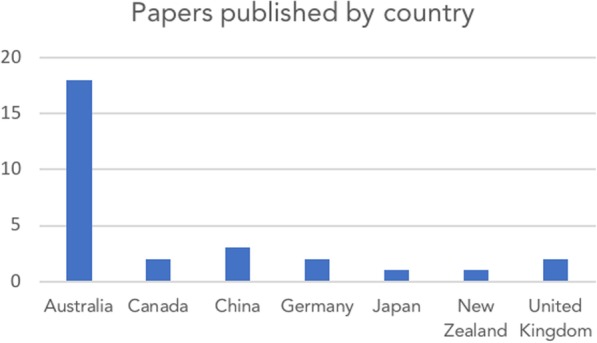
Number of research publications using or citing the WReN Spider by country between 2002 and 2017

Most publications were in English (*N* = 25, 89%), with others in Chinese (*N* = 3, 11%). The first citation of the WReN Spider was in 2006, four years after publication of the original article. Citations peaked in 2015 with 8 (26%) (Fig. 3). Papers came from seven different countries (Fig. 4), Australia generating the most (64%, *N* = 18).

With respect to focus of the relevant articles, 15 (54%) papers used the WReN Spider and 13 (46%) cited it.

### Papers using the WReN spider

The extracted characteristics of the 15 papers using the WReN Spider are shown in Table 1. In 8 cases the instrument was used once to assess the gaps in professionals’ research experience / skills. In 7 cases it was used pre- and post-training to evaluate the impact of the intervention; these interventions were targeted to Allied Health Professionals (e.g, nurses or occupational therapists), General Practitioners and medical graduate students.

**Table 1:** Summary of the 15 papers that used the WReN Spider (in chronological order)

Author & Year of publication	Setting (Institution & Country)	Health professionals	Study design	Type of use ([1]: original Spider alone / [2]: original Spider in conjunction with another assessment / [3]: modified Spider alone/ [4]: modified Spider in conjunction with another assessment)	Used WReN Spider pre and post- intervention?	Main findings
Ried et al., 2006	South Australian Research Network ‘SARNet’, Australia	Network Members (*N* = 89), 32% AHPs, 23% GPs and 45% others	Cross sectional questionnaire survey	[4] WReN Spider used in conjunction with questions in three other areas (personal and professional background, current level of participation in research, and publication and funding record) developed to assess ‘experience in ten core research skills, as well as their interest in developing these skills.	No	Participants reported little or moderate experience in 7 out of the 10 items. ‘Finding relevant literature’ was most often (60%) reported with the highest level of experience. 60% reported no or little experience in ‘Publishing research’ or in ‘applying for research funding’ Participants reported high interest in improving their skills in 9 out of the 10 items. Lower overall interest was reported for ‘finding relevant literature’ and 50% showed ‘high interest’ in ‘analysis and interpretation of results’
Ried et al., 2007	Flinders University, Australia	Primary health care practitioners (*N* = 34) who had received a small research grant	Semi-structured 40 min telephone interview	[2] WReN Spider in conjuction with 6 other questions in similar Spider format relating to the impact of a grant on funding holders capacity, confidence and interest in pursuing research	Yes, measured pre and post intervention of bursaries, grants writers and research fellowships	Median research experience increased for 9 of the 10 skill areas after grant activity
Ried et al., 2008	General Practice Education and Training/ University of Adelaide, Australia	GPs (*N* = 77) who had attended a 3 day research workshop as a GP in training sometime in the previous 5 years	Cross-sectional postal survey	[2] WReN used to measure experience in 10 core areas of research skills as part of a wider questionnaire	Yes, but both pre- and post- assessment measured after the workshop. Pre- based on recall of experience	Self-reported research skills increased over time for the whole group and most significantly for registrars with little or no previous research experience and research project participants. Workshop was reported to have an impact on capacity, confidence and interest in research
Stephens et al., 2009	Healthcare network in an outer metropolitan region of Victoria, Australia	AHPs (*N* = 132) across the network, excluding allied health assistants and AHPs working in mental health	Self-completed paper survey	[4] Research Spider used in the survey as an instrument used to examine clinicians’ level research experience and research interest across 10 core areas	No	AHPs rated themselves as having ‘little research experience’ overall. Although the level of interest was higher than that of experience, items in the WReN Spider specifically relating to research were of little interest to the 85% of them
Short et al., 2009	Emergency department of a major Australian tertiary urban hospital, Australia	Clinical staff in an emergency department (*N* = 67)	Mixed methods evaluation: self completed questionnaire (38 items) followed by focus groups and individual interviews	[4] Questionaire used the 10 items of the WReN Spider for measuring current skills and experience coupled with a repeated WReN Spider assessing participants’ level of interest in developing the 10 core skills	No	The survey including the WReN Spider showed that professionals had limited skills and experience with research. 5 out of 10 items were reported as with “no” to “little experience”. 90% of participants reported needing help to upskill in some of the WReN Spider items. Highest level of interest was that of upskilling ‘analysing and interpreting resutlts’, followed by ‘quantitative research methods’ and ‘critically reviewing the literature’.
Harding et al., 2010	School of Allied Health Professions, Australia	Small group of allied health physicians, first cohort of a 12-week allied health research training (*N* = 7) and their mentors (*N* = 6)	Mixed included in-depth semi-structured interviews and quantitative analysis in research interest, experience, and confidence	[4] WReN Spider used for the quantitative part of the evaluation, measuring research interest, experience and confidence	Yes, both at the onset and at the completion of the program	Confidence in research skills and research experience increased after completion of the program for the first cohort. Conversely, interest in research decreased in 8 of the 10 items of the WReN (all but ‘generating research ideas’ and ‘finding relevant literature’)
Webster et al., 2011	Rural Area Health Services in New South Wales, Australia	Sample of candidates from the 2006 and 2007 cohorts (*N* = 25) of the 2-year long Rural Research Capacity Building Program aimed at developing research skills in rural health workers	Qualitative methods; interview schedule used the capacity building framework to focus the questions for interviewees	[2] WReN Spider used in the first part of the evaluation, assessing changes in experience of candidates in each limb of the WReN Spider. Second part of the evaluation was qualitative aimed at gaining a better understanding of the impacts of the research program	Yes, no specific details provided	Participants valued the program and reported to have gained knowledge and research skills and to have developed research relationships. The WReN identified ‘significant’ improvement (no further detail reported) among candidates.
Leung et al., 2012	Within an academic institution and its affiliated hospitals in Toronto, Canada	Advanced Practice Nurses (APNs, expected to identify and implement research-based innovations and to conduct research to enhance or benefit nursing practice) (N = 9), participating in the Oncology/ Supportive Care Research Mentorship Program	Self-assessment with the WReN Spider and online evaluation	[2] WReN Spider coupled with an online evaluation of each training session and an online survey on the program as a whole.	Yes, prior to the program and at the end of the final education session	Participants felt that their knowledge and experience in research had increased over the course of the program (mean increase in sccore: 0.91). Mentees who had lowest initial scores and who participated the most reported greates improvement. Participants also reported improved leadership skills and increased collaboration and consultation with clients on others in their healthcare teams
Harvey et al., 2013	Queensland Health, a public sector health organisation in northern Australia	Social workers employees of Queensland Health (*N* = 103) providing public healthcare services	Cross sectional survey of social	[4] WReN Spider’s 10 items used in the design of the “experience and need for support in research activities” domain, as part of a wider survey. Research experience assessment included 4 extra items and rating scale from the original Spider was modified.	No	WReN Spider highlighted limited experience and skills in research activities and low confidence levels while participants reported high level of interest. More than 90% reported little/no experience in “applying for research funding” and “publishing research” while highest level of experience were reported for “finding relevant literature” and “critically reviewing literature”
Finch et al., 2013	Organisation providing public healthcare services for the state of Queensland, Australia	Speech language pathologists (*N* = 137) providing public healthcare services	Cross-sectional design study using a customised 30 questions web-based survey	[4] WReN Spider used as part of a survey in a section for health professionals. Used for SPLs to self-rate their level of experience, confidence and interest in each of the 10 WReN research tasks. The final section of the survey asked respondents how many times they had completed each of the 10 research tasks from the ‘Research Spider’ over the last 5 years.	No	Respondents reported higher level of interest than of experience and confidence in research (median interest = 4 ‘moderate’ while median experience = 2 ‘little’and median confidence = 2 ‘little’). Participants more confident and experienced in basic research tasks (“finding relevant literature”) and less confident and experienced in complex tasks (“analysing and interpreting results” and “publishing results”). Only for “finding relevant literature” the level in interest, experience and confidence was the same (5 = ‘very’). For all items non-related to literature, participants reported 1 ‘none’ or 2 ‘little’ experience.
Pighills et al., 2013	Queensland Health Department, Australia	Occupational Therapists (*N* = 86), health practitioners who worked for QHD, 49% hospital based and 51% working in the community or public health	30 min cross-sectional research capacity survey	[4] Questions on research experience based on the categories in the Research Spider. In total the survey instrument had 14 categories, including 10 items from the original WReN Spider	No	The level of support required to become proficient in research activities was inversely related to the level of experience. Experience levels were low in all 14 ares of research. Only in ‘finding relevant literature’ participants reported moderate-to-high levels of experience, but 44% reported little/none experience in it. For the other 13 items, only a quarter or less reported moderate-to-high levels of experience and 93% reported little/no experience in ‘applying fo research funding’. Support needs were lower than experience level only in ‘finding relevant literature’ and in ‘critically reviewing literature’.
Mullan et al., 2014	Graduate School of Medicine at the University of Wollongong, Australia	Three student cohorts of graduating medical students (*N* = 207)	Self-assessment of research experiences using WReN Spider was administered to each student prior to, and the completion	[1] Used alone	Yes, before and after undertaking an individual community-based research project that commenced 2.5 years into the 4-year medical degree program	Reported research experience was higher after program completion in 9 out of the 10 items of the WReN Spider (all but “applying for research funding”, and this was not a component of the curriculum). Significant gains in experience in “writing a research protocol” and “writing and presenting a research report” (rating changed from 1 ‘none’ to 3 ‘some’ and 1 ‘none’ to 4 ‘moderate’) were observed.
Nonoyama et al., 2015	Canadian Respiratory Health Professionals, Canada	Canadian Respiratory Health Professionals (*N* = 119), 77% not in a research-related position while 22% were	Online survey sent via email and monthly e-newsletter to staff developed by the investigators	[4] Experience with and interest in up-skilling were assessed using a simple survey and visually presented using a WReN Spider graph, as part of a wider survey	No	Reported a greater interest in improving the majority of their research skills compared with their level of research experience. All respondents rated their experience as low: no/some experience in 9 out of 10 items of the WReN Spider (all but “finding relevant literature”, rated with “moderate” or “very experienced” by 49%). Interest in upskilling reported as “some” in all 10 areas. Respondents in research positions showed higher interest in developing research skills in all areas but in “finding relevant literature”, compared to respondents in non-research positions.
Pain et al., 2015	Queensland Health, Australia	Queensland health staff classified as Health Practitioners (*N* = 723), 18% from rural areas	Cross sectional survey	[4] WReN Spider’s 10 items used in the design of the “research experience and support needs” domain, as part of a wider survey	No	Rural HPs reported less research experience than metropolitan HPs and need more research support, although the firsr have more qualitative reserch experience. Rural HPs reported low levels of experience in all categories but those related to literature and (finding, reviewing and writing a review). They also reported litte/no experience in “applying for funding” and “writing ethics application”.
Schmidt et al. 2016	Centre for Research Excellence (CRE) in Rural and Remote Primary Health Care Research, Australia	Trainees from 2-year Research Capacity Building Program (RCBP) (N = 8), the trainee’s workplace managers (*N* = 4) and staff of the CRE (*N* = 8)	Survey was conducted using a combination of emailed paper questionnaires and phone surveys	[2] WReN Spider part of a wider survey on the processes and outcomes of the RCBP. Research experience was assessed using the WReN Spider	Yes, baseline WReN Spider completed after an introductory research methods workshop and experience reassessed after program completion	Measurable improvements in self-assessed research experience (average increase 0.6 in average scores). Initially, the group had limited self-rated research experience. All trainees would consider future research and managers thought the RCBP experience was “useful”.

Only one paper was classified as category 1, using the original WReN Spider alone [9]. Three cohorts of graduating medical student used the WReN Spider for self-assessment of research experiences in the ten areas of research activity included in the original instrument. The WReN Spider was administered to each student prior to, and on completion of a community based research project.

Five studies were classifed as category 2, using the original WReN Spider in conjunction with other assessments [10–13]. Ried [10] focused on primary care professionals with research experience to evaluate the impact of a grant funding scheme on skills development using the WReN Spider, during phone interviews. As in Ried [14], professional’s level of participation in research was also assessed. Further, researchers were asked to rate the support intervention and to describe the dissemination of their research findings. Ried [11] conducted a similar study to that of 2007, using the WReN on Australian General Practitioners (GP) registrars having attended the training programme ‘Registrar Research Workshop’ to measure their research experience in the 10 WReN Spider limbs. Other measurements included free text responses to rate the workshop as well as confidence and interest in research with a five-point Likert scale. Webster [15] reported using the original WReN Spider to assess changes in the research experience of rural health workers before and after their candidature in the Rural Research Capacity Building Program. A qualitative evaluation was also conducted, aimed at gaining a better understanding of the impacts of the research program from the perspective of all involved in it. Leung [12] used the WReN Spider as a self-assessment instrument for nurses to enable the different components of a research training program to be tailored to the individual needs. The program’s impact was assessed through online questionnaires after each of the 11 sessions and at the end for the overall program. Each questionnaire encompassed 7 topics (session objectives met, session interesting and relevant, utility of knowledge gained, sharing of information planned, readings helpful, instructor evaluation, satisfaction with session) to be rated on a 5-point Likert scale. Schmidt [13] conducted a survey on trainees from the Research Capacity Building Program (RCBP) using a combination of emailed paper questionnaires and phone surveys. The trainee’s workplace managers and staff of the Centre for Research Excellence in Rural and Remote Primary Health Care Research (CRE) were also surveyed. Trainees self-assessed research experience using the WReN Spider instrument. Baseline assessment was completed after an introductory research methods workshop and was reassessedon program completion. Additional questions on support and supervision aspects of the RCBP were rated on a 5-point Likert scale, such as the manager support, supervision from the program organizer, quality of short course in research methods, peer support and teleconference support.

### No study was classified as category 3, all remaining studies (*N* = 9) fell into category 4

Stephens [16] used the WReN Spider to assess both research experience and interest of allied health professionals (*N* = 132) in Australia. This quantitative data collection was followed by 17 focus groups and individual interviews with participants with the highest level of interest in developing research skills. Data from focus groups and interviews yielded themes within the Dimensional Enhancing Research Capacity (DERC) model.

Ried [14] reported the first use of the WReN Spider amongst primary care professionals since its original publication. In this study, the Spider was used to measure experience but also interest in up-skilling in the ten Spider areas. In addition participants responded to questions about their i) personal and professional background, ii) current level of participation in research and iii) publication and funding achievemnents. Short [17] conducted a mixed-methods study on clinical staff in an emergency department who were asked. All the department members (*N* = 67) completed a 38-item self-evaluation questionnaire utilizing the WReN Spider to evaluate experience in the ten research activities. In addtion, participants were asked about perceived benefits of and barriers to conducting research projects as well as suggestions for future research projects. A repeated WReN Spider was completed to indicate participant’s level of interest in developing each of the ten skills.

Similarily, Nonoyama [18] used the WReN Spider to measure experience plus interest in up-skilling in each of the Spider limbs in respiratory Health Professionals. The questionnaire consisted of closed ended questions, but open-ended questions were used to collect additional comments. Other information covered in the questionnaire regarded demographic information; barriers and facilitators to conducting research; future directions in respiratory research; and research funding and mentorship.

Harding [19] evaluated a 12-weeks research training scheme for allied health professionals. The WReN Spider was used to analyze changes not only in research experience in each of the 10 original limbs, but also in research interest and confidence of those enrolled in the training. In addition, qualitative methods (in-depth semi-structured interviews) were used to explore the experiences of the professionals and their mentors.

Finch [20] used the WReN Spider using a customized web-based survey to study the current levels of interest, confidence and experience performing specific research tasks of speech language pathologists. The survey included three other sections: consent, demographic information and additional research participation questions. In the latter, respondents were asked how frequently they has completed each of the 10 WReN research activities in the last 5 years.

### The WReN spider was extended in three studies [21–23]

Harvey [21] identified the capacity to conduct research in terms of interest, experience, confidence and support needs of social work employees as part of a larger study of health practitioners. Although the survey instrument was designed by the authors, questions on research experience were based on the WReN Spider. The scale was modified for each item from rating each level of experience on a level from 1 to 5 to three-point ordinal scales referring to ‘little/ no’, ‘some’ or ‘moderate/very’ experienced. Moreover, the questionnaire consisted of 14 items. The item ‘Writing & Presenting a research report’ from the original WReN Spider was divided into two items: ‘Writing a research report’ and ‘Presenting research’ and three items were added: ‘Writing a literature review’, ‘Developing a research question’ and ‘Writing an ethics application’.

Pighills [22] surveyed occupational therapists with a questionnaire developed by the authors, including questions on research experience based on the categories in the WReN Spider. Authors added questions on experience of writing a literature review, developing research questions and writing an ethics application. The original item ‘Writing and presenting a research report’ was splitted into two components ‘Writing a research report’ and ‘Presenting research’. The expanded WReN Spider was then used to measure level of research experience and need for support in each of the 14 items. Pighills [22] justified their modifications noting they aimed to make their survery more “specific” and “include other components of the research continuum identified in the literature as areas of limited experience”. The survey developed by the authors also included demographic information, staff self appraisal (practice profile, level of research experience and need for support, perceived barriers and enablers for conducting research, anxiety about conducting research), and open ended questions.

Similarly, Pain [23] developed a survey instrument that included questions on research experience of Health Practitioners based on the WReN Spider. Some modifications to the name of the items were introduced. The authors changed the item ‘Writing a protocol’ to the more generic item ‘Writing proposals’ and the item ‘Analysing and interpreting results’ to simply ‘Analysis’, ‘Finding relevant literature’ was changed to ‘Finding literature’ and ‘Critically reviewing literature’ to ‘Reviewing literature’. Like Harvey [21] and [22], the authors split ‘Writing and presenting a research report’ in two items, ‘Report writing’ and ‘Presenting research’. Finally, three items were added: ‘Writing a literature review’, ‘Developing questions’ and ‘Writing ethics’. This group did not provide a rationale for the amendments to the original WReN Spider. This modified 14-items Spider was used for self-rating of experience and support needs. Additional questions in the online survey assessed factors influencing research engagement and participation and barriers and enablers of it.

### Papers citing the WReN spider

#### Thirteen papers (46%) cited the WReN spider

Of the papers just citing the Spider five were from Australia, 3 from China and 2 from Germany. Holden [24], Lazzarini [25], Williams [26], Pan [27], Gong [28] and Wu [29] cited the original paper when citing contemporary instruments measuring participants’ self-rated individual research experience. These cite the fact that the WReN only includes the individual domain as a shortcoming. Janssen [30], Huber [31] and Williams [26] cited the original Spider publication in a list of published evaluation studies of research training programmes. Borkowski, [4] cites the WReN as a tool to measure research experience used in some of the studies included in their review. Holden [32] consulted the items in it when developing the individual level domain of the Research Capacity and Culture (RCC) tool. Eam [33] mentioned the WReN Spider as one of the tools consulted when developing their own instrument to measure research involvement among faculty members. Huber [2] cited the items in the WReN Spider as a way to operationalize their review of instruments to measure individual’s research experience.

These papers related to the evaluation of interventions targeted to Allied Health Professionals (*N* = 5, 18% of the total number of studies) and nurses (*N* = 3, 11% of the total number of studies) followed by podiatrists (*N* = 2, 6% of the total number of studies). The rest of the studies aimed to assess training of specific health professions of faculty members and medical graduate students.

Five papers [24–26, 32, 34] employed the validated RCC instrument, validated in Holden [32], that allows measurement of research capacity and culture at organization, team and individual levels with robust scale items. Participants rate each of the 50 items on a 10-point scale (with one considered as the lowest skills or success level and ten the highest possible skill or success level), and items are scored separately for each domain with robust scale items examining three domains. The individual domain was partly based on the ‘WReN Spider’, and items for the other domains were based on previous literature. Out of the 5 studies using the RCC, 4 were published in Australia and 1 in the UK.

Two studies used other validated instruments, the Edmonton Research Orientation Survey (EROS) and the Estabrook’s Research Utilisation Questionnaire and the Research Self-Efficacy Scales (RSES) and Scholarly Activity Scale (SAS). Janssen [30] combined qualitative interviews with different measurement instruments (the EROS and the Estabrook’s Research Utilisation Questionnaire) to measure change in attitude to and capacity of research in allied health professionals working in the acute care setting. Gong [28] used the RSES and SAS to rate the efficacy of research training for Master and PhD students.

Four papers used self-reported survey questionnaires with about 50 items, always coupled with open questions capturing participants’ comments and recommendations. Huber [31] aimed to validate the Local Health Research Capacity Strengthening (LHRCS) in four different training settings in Tanzania on either clinical research skills or on clinical skills necessary for research projects. The questionnaire, that was based on a theoretical model, was modified after the intervention to include four subscales with 19 items, three global impression items and open questions for participants’ comments and recommendations. Eam [33] used self-reported survey questionnaires in 10 Cambodian universities, containing 47 key items organized into three sections, exploring how individual factors and institutional factors affect involvement in research activities. The items in this last section were partly based on the activities included in the WReN Spider, with some modifications done on the language to ease understanding in the Cambodian academic context. Pan [27] used a survey to revise scientific research ability of nurses with self-evaluating rating scales with 37 items in 6 categories: academic writing capability, document analysis capability, capability to conduct research, capability to design research, capability to do literature review, capability to identify research problems. The survey was designed by doctors and matrons and initially included 50 items, that were reduced to 37 after a pilot with nurses. Wu [29] followed a similar approach and consulted with experts and matrons to formulate 36 items for survey, based on their experience and on a literature review. The 35 items finally included were divided into 5 categories: papers and research projects, research awards and results, capability to conduct research, capability to identify problems and professional training, communication capability.

Huber [2] conducted a systematic review to optimize and systemize future efforts in the Health Research Capacity Development (HRCD) field by providing overview of the Needs assessment, Monitoring and Evaluation (NaME) activities at the individual and organizational level with a focus on methods, instruments and instruments used. This review used the WReN Spider to operationalize the “Research” category of NaME framework used to assess each instrument. The review concluded there was a need for a coherent and transparent taxonomy of Health Research Capacity Development (HRCD) to maximize the benefits of future studies in the field.

Borkowski [4] conducted a systematic review on the research culture of allied health professionals. The review found that different instruments to measure research capacity and culture were used in the original studies reviewed, including the RCC instrument and the WReN Spider, and suggested that collaborative efforts with external partners and research leadership were needed to intensify allied health professionals research culture and capacity.

## Discussion

The ‘Wessex Research Network (WReN) Spider’ is an instrument developed and validated 20 years ago to assess research experience of members of a UK Primary Care Research Network. The network had been established to stimulate research awareness and involvemement amonst health care workers in General Practices. This bibliometric study, tracing the use and development of the instrument, found 31 citations of the WReN Spider in the published literature. Of these 18 were studies which have used the Spider to assess the training needs of health professionals or were before and after studies to evaluate the impact of training initiatives. The WReN Spider has been most used in the areas where activites to grow research capacity are in the early stages of development, such as nursing and allied health professions [35–38]. Its use in Australia may be because its developer promoted its use at a national conference in 2001, when Primary Care research initiatives were in their infancy [39–41]. We contacted the authors of these studies who described the characteristics of the WReN Spider most valued; these characteristics were its relevance and being a “straight-forward and brief tool to use”. Whilst the WReN Spider continues to be used, it is frequently enhanced with the use of additional questions that explore the wider issues of research success, including collaborators, resource and environment and also with a qualitative component to the evaluation. Some adjustments have been made to the research activities represented in the legs of the Spider.

The WReN Spider inaugural paper presented data supporting the validity of the instrument in the setting in which it was used. Interestingly none of the subsequent studies have repeated a validation process in their local context, with perhaps cultural and linguistic differences. This may have been for purely pragmatic reasons, as validation studies are often unfunded and there may not have been the resources available. Another factor might be the misunderstanding generated by the shorthand we frequently use when describing measurement instruments. The tendency is to talk about instruments being ‘validated’ rather than the measurements generated by the instruments which are considered valid. If it is really the measurements to which claims of validity pertain then it becomes apparent that validation is a continuous process rather than a one-off activity. From a pure psychometric point of view, validation needs to be repeated in each new setting or population. Interestingly, the WReN Spider has only been used in anglophone countries. Often the need to linguistically validate a measurement instrument (i.e. to translate it rigorously in order to ensure linguistic equivalence) triggers awareness regarding psychometric validation also. This lack of awareness of the necessity for ongoing validation and a paucity of resources (time, staff, money, skills) means it will remain an often neglected area.

There is very little direct critique of the WReN Spider structure with the five point scale on ten legs. Short [17] highlights the potential risk of reports with a high rate of missing data, with as many as 12% of the participants not completing the 10 items.

Missing data may reflect that there is no option for the respondent to indicate that experience in the research area being assessed is not relevant to them. For example, the WReN Spider measures experience both in qualitative and in quantitative research, while many researchers may focus their activities on one or the other methodology. Similarly the assessment of two activities on a single scale (for example ‘writing and presenting a research report’, and ‘analysing and interpreting results’) may discourage a respondent completing the scale if they have divergent experience of these two discrete skills. Further, the role of social desirability bias arising from the desire of the respondent not to appear inexperienced is unclear. It may result in missing data or data skewed to the positive end of the response spectrum.

The WReN Spider structure has been maintained in the 10 studies that measure constructs beyond experience, including confidence or interest in up-skilling in each of the 10 items. Many of the shortcomings of the WReN Spider are expressed implicitly in the manuscripts, through the changes, additions and modifications made by other researchers to the instrument. Qualitative components added to the questionnaire have enabled a better understanding of the barriers and motivators for individuals undertaking research and the specific impact of interventions (research training and funding) on research activity.

Already we have a WReN Spider with ten appendages which challenges the physical characteristics of an arachnid with four pairs of legs, but as the research culture becomes more sophisticated we may need more legs to represent activites such as good clinical practice (GCP), good research practice and patient and public involvement (PPI) in research. As Pighills [22] reminds us, the WReN Spider is an interim or intermediate measure of success because “traditionally impact is measured by grants acquired and peer-reviewed articles published, rather than experience or skills”. The WReN Spider will continue to be a process measure, but the move away from grant income and bibliometric prowess as measures of success to valuing the health and health sector benefits of research is to be welcomed. However, it calls for the addition of yet another Spider leg, one that addresses the activities linked to the preparation of case studies summarising impact.

The original WReN Spider focused on the individual, but research is becoming much more of a team activity and a multidisciplinary activity. The need for a more holistic approach to improving the research culture of health professionals is now recognised and individuals need to be considered as part of a team and an organization. Cheetham [42] defined research culture as the “*structure that gives [research behaviour] significance and that allows us to understand and evaluate the research activity*.” Research culture cannot be developd merely by addressing the skills and experiences of the individual, but needs to focus on the individual and the organisation synergistically [43]. This inclusive vision of research activities has been addressed in the Australian Research Capacity and Culture (RCC) instrument which measures the research skills of individuals together with the research team’s and the organization’s capacity to support research. The RCC instrument, with its multidimensional approach to improving research culture, also provides an opportunity for participants to determine their perceptions of their team’s and organisation’s research skills. As health research becomes more integrated into industry and health care there may be a need for a supra-organisational domain to be developd to capture these activities.

## Data Availability

All data generated and analysed during the current study are included in this published article.

## Abbreviations

CRE: Centre for research excellence
DERC: Dimensional enhancing research capacity
EROS: Edmonton research orientation survey
GCP: Good clinical practice
GP: General practitioners
HRCD: Health research capacity development
LHRCS: Local health research capacity strengthening
NaME: Needs assessment, monitoring and evaluation
PPI: Patient and public involvement
RCBP: Research capacity building program
RCC: Research capacity and culture
RSES: Research Self-Efficacy Scales
SAS: Scholarly activity scale
WReN: Wessex Research Network
